# Psychological Network Analysis of General Self-Efficacy in High vs. Low Resilient Functioning Healthy Adults

**DOI:** 10.3389/fpsyt.2021.736147

**Published:** 2021-11-17

**Authors:** Katja Schueler, Jessica Fritz, Lena Dorfschmidt, Anne-Laura van Harmelen, Eike Stroemer, Michèle Wessa

**Affiliations:** ^1^Department of Clinical Psychology and Neuropsychology, Institute of Psychology, Johannes Gutenberg-University of Mainz, Mainz, Germany; ^2^Medical Informatics Group, University Hospital of Frankfurt, Frankfurt, Germany; ^3^Department of Psychiatry, University of Cambridge, Cambridge, United Kingdom; ^4^Institute of Education and Child Studies, University of Leiden, Leiden, Netherlands; ^5^Leibniz Institute for Resilience Research Mainz, Mainz, Germany

**Keywords:** resilience, network analysis, self-efficacy, connectivity, partial least squares regression

## Abstract

Resilience to stress has gained increasing interest by researchers from the field of mental health and illness and some recent studies have investigated resilience from a network perspective. General self-efficacy constitutes an important resilience factor. High levels of self-efficacy have shown to promote resilience by serving as a stress buffer. However, little is known about the role of network connectivity of self-efficacy in the context of stress resilience. The present study aims at filling this gap by using psychological network analysis to study self-efficacy and resilience. Based on individual resilient functioning scores, we divided a sample of 875 mentally healthy adults into a high and low resilient functioning group. To compute these scores, we applied a novel approach based on Partial Least Squares Regression on self-reported stress and mental health measures. Separately for both groups, we then estimated regularized partial correlation networks of a ten-item self-efficacy questionnaire. We compared three different global connectivity measures–strength, expected influence, and shortest path length–as well as absolute levels of self-efficacy between the groups. Our results supported our hypothesis that stronger network connectivity of self-efficacy would be present in the highly resilient functioning group compared to the low resilient functioning group. In addition, the former showed higher absolute levels of general self-efficacy. Future research could consider using partial least squares regression to quantify resilient functioning to stress and to study the association between network connectivity and resilient functioning in other resilience factors.

## Introduction

Stress resilience describes the maintenance or quick recovery of mental health during or after adverse events (1, 2). Resilience research is part of a shift in perspective from a focus on psychopathology to the strengthening and maintaining of mental health. This is of great importance for preventing and overcoming the consequences of stress–as leading mental health organizations have already pointed out (3–5). A promising research method in this context could be psychological network analysis (6), a method that has advanced our understanding of psychopathology (7) and that has more recently been introduced to resilience research: for example, studies that examined the links between multiple resilience factors (e.g., optimism, social support, self-efficacy) at a fixed point in time (static resilience factor networks) or studies that model associations between stressors, resilience factors, and other factors over time [dynamic hybrid symptom-and-resilience-factor networks; (2, 7–10)].

The present study builds upon these previous results and investigated the network structure of one resilience factor in particular, i.e., general self-efficacy. This means, we do not include psychopathology symptoms or other external factors in our networks. Instead, we investigate the network connectivity of general self-efficacy in two groups that differ in their level of resilient functioning. Resilient functioning to stress is therein conceptualized as the participants' degree of mental health, as indicated by well-being and psychopathology symptoms, in relation to their experienced stress history (11). This conceptualization represents the current view of resilience as the result of a successful adaptation to daily stressors and life events (including traumatic experiences) (2, 12, 13). We assume that higher network connectivity of self-efficacy reflects increased protection against adversity, because several attributes of self-efficacy are activated simultaneously when dealing with stress. As a result, we believe that stronger connectivity is also associated with higher resilient functioning. In the following we elaborate in detail for the reader the role of self-efficacy and network connectivity in the scope of resilience research.

### Self-Efficacy as Resilience Factor

Resilience factors are considered as promoters of stress resilience. They might come as external resources such as family or friendship support (14). Further, they might emerge on an intra-individual level, for instance as self-esteem, emotion regulation skills (15), self-compassion (16) and self-efficacy (17).

Self-efficacy refers to the believe in one's ability to “execute actions required to deal with prospective situations” (18, 19). It can be of general or context-specific manner, the latter e.g., with respect to coping abilities with severe diseases, parenting or caregiving, academic, or leadership performance. Although some research also hints to potential costs of high self-efficacy particularly with respect to performance (20, 21), high self-efficacy has shown to have a buffering effect on the detrimental impact of adverse events and daily stressors on mental health [e.g., (21, 22)]. For instance, when facing traumatic events, perceived coping self-efficacy served as mediator of posttraumatic recovery (23) and mitigated PTSD symptoms (24) after generally traumatizing events as well as single devastating events (25, 26). High self-efficacy is thus considered as a resilience factor, i.e., facilitating resilient functioning (1, 27). However, very little is known about the network structure of self-efficacy (28), which would allow elucidating on the interplay between different aspects of self-efficacy in facilitating resilience. As such analytical approach has brought forward pathogenetic research (7), the present study proposes to examine self-efficacy as resilience factor network.

### Connectivity in Psychopathology and Resilience Factor Networks

Networks are graph-based representations of complex systems in the real world (29). It is a common application of psychological network analysis to model symptoms of mental disorders (7). Often, such networks include psychopathology symptoms as nodes and their interrelations as edges. For network connectivity, as one indicator of network analyses, a simulation study showed that stronger connectivity, i.e., higher interrelations between network nodes, indicated worse prognosis of major depressive disorder (MDD) (8). The authors used a pictorial example to illustrate the role of connectivity: strongly related symptoms could be seen as closely related domino blocks. When one domino block falls over, other blocks would easily fall over as well. That is the activation of one MDD symptom increases the likelihood that other symptoms will also be activated. Overall, this mechanism makes the network more likely to switch into a depressed state and more di?cult to reverse into a healthy state. Empirical studies have supported these findings, with stronger connectivity in symptom networks of depression being associated with more severe symptoms and worse prognosis 2 years later (30) as well as with poor treatment response in depressed adolescence (31). Thus, the strength of connectivity in psychopathology symptom networks seems related to the severity of mental illness.

How this mechanism exactly behaves in the context of stress resilience is still unclear and our study only contributes a puzzle piece to answer this question. On a theoretical level, we suggest that the role of connectivity in psychopathology networks is inversely mirrored in self-efficacy and other resilience factors. Strong connectivity would then be associated with increased effectiveness of the resilience factor. A person with high self-efficacy connectivity might be better protected against adversity und thus function more resiliently. In essence, this might be because several attributes of self-efficacy are simultaneously activated and positively enhance each other. For instance, when faced with a severe problem, not only the problem-solving part of self-efficacy, but also other attributes, such as confidence in one's coping mechanisms or one's ability to pursue goals, are activated for dealing with the stressor. We presume that this will be reflected in higher resilient functioning to stress. Psychological network analysis provides an appropriate method for investigating such theoretical considerations.

### Aim of This Study

Our hypotheses are inspired by aforementioned findings from psychopathology symptom networks, which suggest that stronger connectivity in symptom networks is related to more severe psychopathology.

We hypothesize that stronger connectivity in self-efficacy networks should be associated with higher resilient functioning to stress. We investigate this statement by comparing self-efficacy network connectivity between a high resilience and a low resilience group based on resilient functioning scores. To calculate resilient functioning scores, we extend the so-called residual approach (11) by means of partial least squares regression (32). Regarding the network analysis of self-efficacy, we expect higher network connectivity in the high resilience group than in the low resilience group.

## Methods

### Sample

Participants were 875 (73% women) undergraduate students (*M* = 21.97, *SD* = 3.86) taking part in a longitudinal study investigating the effects of a blended resilience training. In the present study, baseline self-report data from all participants (irrespective of later group membership) collected between October 2017 and May 2019 were included. Exclusion criteria for study participation were any psycho-pharmacological medication, alcohol abuse, severe traumatic events (severe car accident, being sent to war, being held hostage, life-threatening natural disaster, sexual violence, or serious robbery), self-harm or suicidality, and psychotherapy during the last 5 years.

### Measures

We used self-report measures to assess exposure to minor and major potentially stressful events, mental health, general self-efficacy, and demographic information. All instruments were administered in German.

#### Checklists of Minor and Major Stressors

We used two checklists to assess individual exposure to minor and major stressors. Stressors refer to any event or circumstance that could be perceived as especially stressful.

The Daily Hassles Checklist (DH) is a self-report of daily or minor stressors (33). It assesses frequency and severity of 58 different minor stressors during the last seven days by asking a) “On how many days did the event occur?” (number of days from 0 to 7) and b) “How stressful was the event for you?” on a scale from 1 (“not at all”) to 5 (“very much”). Examples of minor stressors are “problems due to lack of help from others,” “commuting,” or “nightmares.”

The Life Events Checklist [LE, as used in Chmitorz et al. (34); adapted from Canli et al. (35)] assesses life-time exposure to 27 major stressors. Participants indicate whether or not the event happened and, if so, how stressful they experienced it on a scale from 1 (“not at all”) to 5 (“very much”). Examples for major stressors are “death of a relative,” “sexual abuse,” or “job loss.” A complete list of all minor and major stressors can be found in the Supplementary Material.

#### Mental Health Questionnaires

Two self-report questionnaires served as indicators for current mental health: (a) the World Health Organization Well-Being Index, WHO-5, (36) and (b) the Brief Symptom Inventory of psychopathology symptoms, BSI-18 (37, 38). The 5-item World Health Organization Well-Being Index is a brief and popular questionnaire for mental well-being that has been translated into more than 30 languages (36, 39). Ratings of statements such as “I have felt cheerful and in good spirits” over the last 2 weeks are assessed on a scale from 0 (“At not time”) to 5 (“All of the time”). All items avoid clinical symptom-related language and focus primarily on positively phrased questions. The German version of the WHO-5 demonstrates very good reliability with Cronbach's α = 0.92 (40). The German version of the Brief Symptom Inventory, BSI-18, is a short version of the Symptom Checklist SCL-90 (41, 42). The scale consists of 18 psychopathology symptom items. Symptoms such as “loneliness” or “thoughts about death and dying” were rated on a scale from 1 (“not at all”) and 5 (“very much”) with respect to how often they bothered the participant during the last 7 days. Six items each were combined to three distinct subscales: anxiety, depressive, and somatic symptoms. All subscales achieve good reliability indicated by Cronbach's α = 0.82 for somatization, α = 0.87 for depression, and α = 0.84 for anxiety (38). To facilitate interpretation, we inverted the scores of all three BSI-18 subscales; that is, higher values indicated lower levels of each anxiety, depression, and somatic symptoms.

#### Self-Efficacy Questionnaire

The General Self-efficacy Scale (43, 44) served as a measurement of self-efficacy expectancy. This unidimensional 10-item scale has demonstrated good reliability, with Cronbach‘s α ranging from 0.76 to 0.90. Participants indicate on a scale from 1 (“not true”) to 4 (“very true”) how strongly they agree with general statements such as “I can always manage to solve di?cult problems if I try hard enough.” We used the German version of the General Self-efficacy Scale (see http://userpage.fu-berlin.de/~health/selfscal.htm to access the questionnaire in 32 languages).

### Statistical Analysis

Summarizing the statistical procedure, we first calculated resilient functioning scores. Mental Health was indicated by participants' ratings of the BSI-18 and WHO-5. Stress history was assessed through LE and DH. Since resilience itself is a dynamic concept it cannot be measured cross-sectionally. However, it is possible to calculate the degree of resilient functioning to stress at a certain time point as a proxy to resilience. The present measure of resilience, i.e., resilient functioning to stress, results from relating LE and DH (indicators of stress history) to BSI-18 and WHO-5 (as indicators of mental health). In this, we used partial least squares regression (PLSR) to predict mental health from stress history. From the PLSR model we extracted regression residuals which further served as resilient functioning scores. Based on these values we divided our sample into a high resilience and a low resilience group. For both groups we then performed psychological network analysis on GSE data. We calculated self-efficacy networks and compared global connectivity metrics between the groups. Additionally, we compared absolute levels of self-efficacy, i.e., GSE item ratings and GSE total score, between both groups. More details follow in the next sections.

#### Data Pre-processing

We prepared raw data for further analyses as follows. Four dependent variables were prepared for PLSR. We calculated a WHO-5 sum score and aggregated BSI-18 items to three BSI-18 subscale scores. The latter were inverted to facilitate interpretation in line with WHO-5 scores. This resulted in four continuous indicators of current mental health with higher values indicating high subjective well-being, low anxiety symptoms, low depressive symptoms, low somatic symptoms. Next, we z-standardized and normalized these scores and regressed out the effect of age and gender. Second, 147 independent variables were prepared for PLS regression. DH and LE items both included values below 0 on item level meaning that the corresponding event either did not happen at all or the participant did not wish to reply. All these values were set to exactly 0.

#### Stress Resilience Scores

There is no established gold standard for quantifying stress resilience (1, 6). One promising approach is to measure resilient functioning as doing better than expected given one's history of stress exposure (11, 45). van Harmelen et al. (11) quantified resilience in a two-step approach, where first Principal Components Analysis is used to collapse stress exposure and mental health into one dimension, respectively. Subsequently, the residuals of the regression of the summary mental health score on the summary stressor scores indicate the resilient functioning of mental health to stress. We combined these formerly two independent steps into one comprehensive model using a powerful statistical method, partial least squares regression (PLSR) (32, 46). To calculate stress resilient functioning scores a one component PLSR model has been applied. DH and LE were predictors and four mental health indicators (WHO-5, inverted BSI-18 subscales) were outcome variables. The amount of variance in outcomes explained by the model was statistically significant compared to 1,000 permuted random models, i.e. it explains more variance in outcomes than expected by chance (*p* < 0.01). For each subject, we extracted the mean PLSR prediction residuals as resilient functioning scores to stress.

#### Sample Split

Next, we split our sample into two groups and used 0 as the criterion for allocating the participants. The first group, the highly resilient group, consisted exclusively of participants who had a resilience score above 0. The second group, the low resilient group, included only participants with a resilience score ≤0.

#### Network Analysis

In both groups we calculated cross-sectional partial correlation networks of self-efficacy. Nodes of each network were the ten GSE questionnaire items. Network edges were partial correlations regularized using the least absolute shrinkage and selection operator (LASSO) method. These were computed as polychoric correlations which are suitable for ordinal data. The LASSO algorithm was used to regularize edges. This results in a rather sparse network by setting edges close to zero to exactly zero and hence exclude them from the network. We computed the following global network measures: (a) average strength of all nodes, (b) average expected influence, and (c) the average shortest path length (9, 47). **“Average strength of all nodes”** is a global measure for the entire network and results from averaging the node strengths of all individual nodes. The strength of a single node is defined as the sum of all edges (absolute values) that are directly related to the node at hand, thus, the sum of all the direct interrelations the node at hand has with other nodes in the network. The assumption is that node strength indicates how strongly a node is directly connected to other nodes in the network. **“Expected influence”** is a similar metric as node strength, with the only difference being that expected influence takes the sign of the edges into account. In other words, if an edge is negative it gets subtracted from and if it is positive if gets added to the other edge parameters of the node at hand. To get a global metric we then again averaged over the expected influence scores of all the individual nodes in the network. **“Average shortest path length”** is defined as the mean edge weight of the steps along the shortest paths for all possible pairs of network nodes. For example, the shortest path length of two specific node gives an indication for how long it takes to traverse the network from the first to the second node (9, 47). It is important to point out that all of those metrics are group metrics, so they are averages over all people who are in the network and do not necessarily generalize to every single individual in the group.

In addition, we used the network comparison test (NCT), a permutation-based method that allows significance testing of global network strength (30, 48). Even though NCT is the state-of-the-art of network comparison tests, it has not yet been validated for ordinal data. This limits the interpretation of NCT test results in our study.

#### Software

We used the programming language R 3.5 (49, 50), the IDE RStudio 1.14, and Matlab 2018b (The MathWorks Inc.) for statistical analyses. Matlab PLS regression code was adapted from publicly available work by Whitaker et al. (46). R code for network analysis strongly builds on the R packages “bootnet” and “qgraph.” Corresponding tutorials are provided by Epskamp et al. (51) and Epskamp and Fried (52). Resources for the “NCT” R package is provided by van Borkulo (48) and van Borkulo et al. (30). All analysis scripts necessary to replicate the analysis of the present study will be available online upon peer-reviewed publication (https://osf.io/pz5ky). Raw and processed data are available upon request.

## Results

Based on the resilient functioning score, we divided the total sample into two groups. Participants in the *high resilient functioning group* (*n* = 468, 74% women) had a resilience functioning score above 0. This means that all of them showed fewer psychopathological symptoms and higher well-being than statistically expected based on their experienced adversity (daily hassles and life events). Participants in the *low resilient functioning group* (*n* = 407, 72% women) had a resilient functioning score of 0 or less. This means that all of them showed as many or more psychopathological symptoms and lower or equal well-being than statistically expected based on their experienced adversity.

### Differences in Resilient Functioning to Stress

As a sanity check, we tested expected differences in resilient functioning between both groups. A *t*-test confirmed that the two groups significantly differed in their resilience scores with a large effect size (*t* = 37.65, *p* < 0.001, Cohen's *d* = 2.58). The highly resilient group (*M* = 1.81, *SD* = 1.41) scored significantly higher in stress resilience than the low resilient group (*M* = −2.09, *SD* = 1.62). See Table 1 for details. This confirms that the group split procedure had worked as expected.

**Table 1:** Characteristics of and differences between both groups.

	**High resilience**	**Low resilience**	**Difference**
Group size	*n* = 467	*n* = 408	
Gender (female)	*n* = 344 (74%)	*n* = 294 (72%)	
Age	*M =* 21.76 (*SD* = 3.42)	*M =* 22.20 (*SD* = 4.3)	n.s.
Σ minor stressors (freq)	*M =* 68.81 (*SD* = 32.76)	*M =* 65.12 (*SD* = 28.33)	n.s.
Σ minor stressors (sev)	*M =* 61.88 (*SD* = 30.58)	*M =* 60.46 (*SD* = 26.53)	n.s.
Σ major stressors	*M =* 19.84 (*SD* = 16.40)	*M =* 20.02 (*SD* = 15.49)	n.s.
Σ GSE	*M =* 59.88 (*SD* = 7.79)	*M =* 54.45 (*SD* = 7.93)	^*^
GSE 01	*M =* 3.25 (*SD* = 0.53)	*M =* 2.99 (*SD* = 0.52)	^*^ *^c^*
GSE 02	*M =* 3.29 (*SD* = 0.56)	*M =* 3.10 (*SD* = 0.57)	^*^ *^c^*
GSE 03	*M =* 2.91 (*SD* = 0.67)	*M =* 2.59 (*SD* = 0.72)	^*^ *^c^*
GSE 04	*M =* 2.55 (*SD* = 0.63)	*M =* 2.28 (*SD* = 0.74)	^*^ *^c^*
GSE 05	*M =* 2.96 (*SD* = 0.58)	*M =* 2.68 (*SD* = 0.65)	^*^ *^c^*
GSE 06	*M =* 2.73 (*SD* = 0.75)	*M =* 2.32 (*SD* = 0.76)	^*^ *^c^*
GSE 07	*M =* 3.13 (*SD* = 0.70)	*M =* 3.01 (*SD* = 0.77)	^*^ *^c^*
GSE 08	*M =* 3.11 (*SD* = 0.68)	*M =* 2.85 (*SD* = 0.75)	^*^ *^c^*
GSE 09	*M =* 2.81 (*SD* = 0.59)	*M =* 2.56 (*SD* = 0.59)	^*^ *^c^*
GSE 10	*M =* 3.02 (*SD* = 0.56)	*M =* 2.84 (*SD* = 0.56)	^*^ *^c^*

*n.s., not significant with p > 0.05*;

* *p < 0.001*;

c *bonferroni corrected p-values*.

### Differences in Absolute Levels of Self-Efficacy

**Figure 1:**
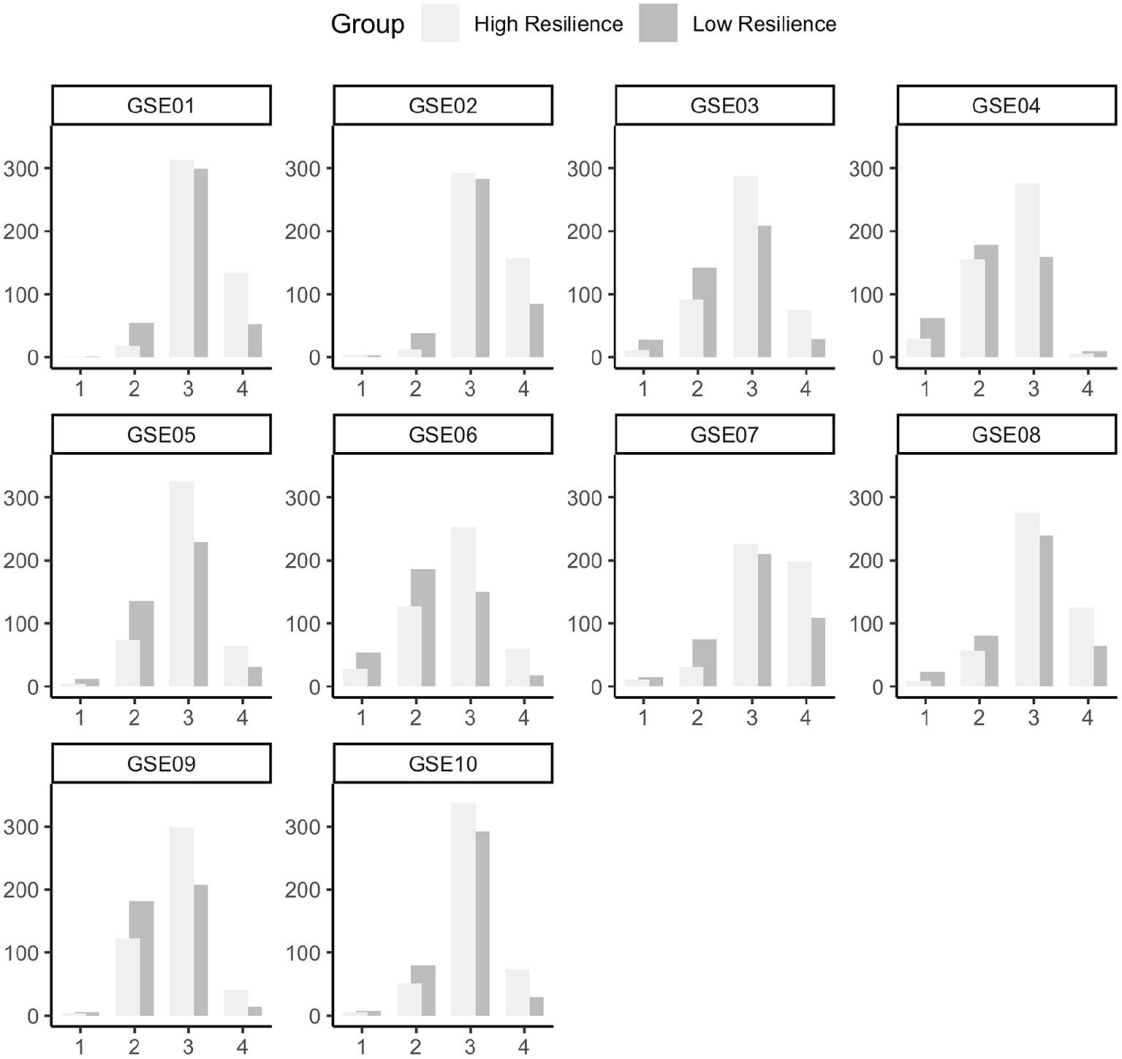
Data distribution of all ten items of the general self-efficacy scale (43). Abbreviations GSE01 until GSE10 indicate item numbers. The x-axis shows the response ranging from 1 (“not at all”) to 4 (“very much”) and frequencies, i.e., number of participants, are depicted on the y-axis.

We compared absolute levels (total and per item scores) of self-efficacy between the groups using a *t*-test and Wilcoxon rank sum tests. The GSE total score was higher in the highly resilient group (*M* = 30.0, *SD* = 3.9) than in the low resilient group (*M* = 27.2, *SD* = 3.9). This difference was statistically significant with a medium effect size (*t* = −10.3, *p* < 0.001, Cohen's *d* = −0.7). To test for group differences in GSE items we used 10 Wilcoxon rank sum tests (two-sided, Bonferroni corrected). Across all GSE items, highly resilient adults had significantly higher levels of self-efficacy than low resilient adults (all corrected *p* < 0.001). See Figure 1 for general self-efficacy differences.

### Connectivity of Self-Efficacy Networks

**Figure 2:**
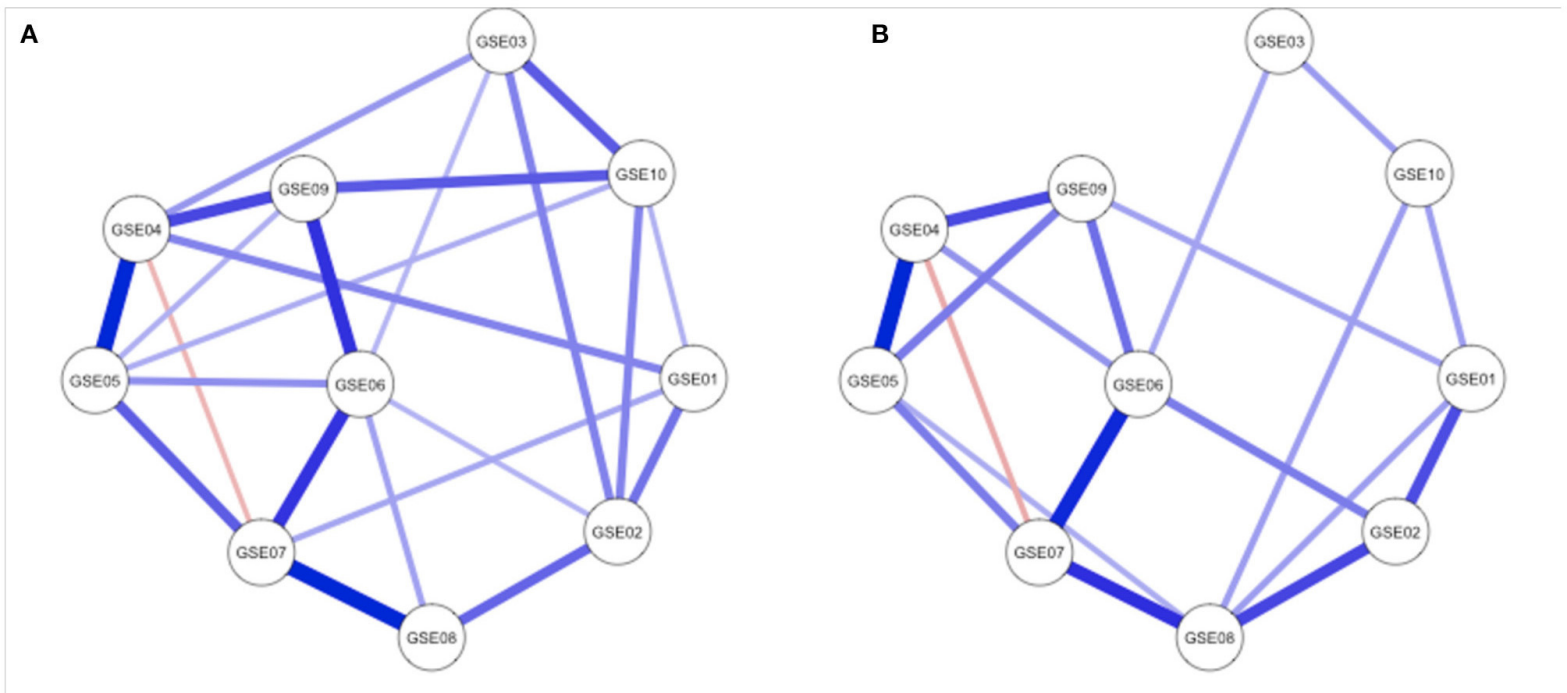
Regularized partial correlation networks of **(A)** the high resilient functioning group (*n* = 467) and **(B)** the low resilient functioning group (*n* = 408). Network nodes refer to the items of the General Self-Efficacy Scale (43). Edges show regularized partial correlations between all ten questionnaire items (GSE01 until GSE10). A thicker line indicates higher correlations. No line indicates that the correlation between this respective pair of nodes was did not survive regularization.

We expected three global network measures to differ between high and low resilient participants: average node strength, average node expected influence, and average shortest path length. As described in the methods section, these network measures indicate connection strength and distances between GSE items (nodes). As expected, strength and expected influence were higher in the high resilience group and shortest path length was lower in the high resilience group compared to the low resilience group (see Table 2). Connectivity was thus higher in the self-efficacy network of the high resilience group than in the self-efficacy network of the low resilience group. The group with higher resilient functioning scores is characterized by stronger connections between the individual attributes of self-efficacy (GSE items), e.g., trust in problem solving, belief in coping skills, or ability to pursue goals. The Network Comparison Test confirmed differences in global strength on trend level, yet not significant (*P* = 0.06), with the limitation that this test has not been validated for ordinal data, only for binary and Gaussian data. Figure 2 shows the self-efficacy network of both groups. Network parameters remained qualitatively similar when based on unthresholded LASSO-regularization and on unregularized partial correlations (see Supplementary Figures S1, S2).

**Table 2:** Global connectivity of self-efficacy networks.

	**High resilient functioning**	**Low resilient functioning**
Strength	0.88	0.77
Expected influence	0.84	0.72
Shortest path length	7.43	9.07

*Comparison of global network connectivity measures in high resilient (n = 467) compared to low resilient (n = 408) adults. Values indicate the average connectivity parameters per node*.

## Discussion

This study examined the global connectivity of a specific resilience factor network, i.e., self-efficacy. We compared self-efficacy networks between two groups: one that is high in stress resilience and one with low stress resilience. As expected, our analyses show in three network metrics that the high resilience group has stronger connectivity in their self-efficacy networks than the low resilience group. In addition, the high resilience group showed higher self-efficacy scores when compared to the low resilience group. To summarize, high resilience was associated with general self-efficacy whose single parts are more densely connected (from the network perspective) and have higher ratings (from the perspective of summed questionnaire scores). Our results provide considerable evidence to include network connectivity in further research on resilience factors.

### Contributions

To the best of our knowledge, this is the first study to relate network connectivity of resilience factor networks to an estimate of overall resilience. Our results contribute to resilience research in several ways. We provide evidence that network connectivity between attributes of a resilience factor, here general self-efficacy, and general stress resilience are inter-related (note: resilience factor networks and resilience scores were independently computed).

Network connectivity has been shown to play an important role in psychopathology symptom networks (8, 30, 31). It has been discussed as potential prognostic indicator for treatment response in major depression (30, 31). Because the present study is based on cross-sectional data, we cannot make any statement regarding the prognostic value of connectivity in resilience factor networks. In practical terms and in analogy to previous results of depression research (7), our results could still provide a hint that in highly resilient people different attributes of self-efficacy are more interconnected, which could potentially enhance the combined effectiveness of the different facets of this resilience factor.

Moreover, our findings underpin the importance of self-efficacy as resilience factor, because the high resilience group reported higher self-efficacy than the low resilience group. This is in line with previous research showing that self-efficacy serves as buffer against potentially traumatic and daily stressors (22, 24).

Finally, the present study features promising research methods for the investigation of stress resilience and resilience factors. Our methodological approach displays a promising measure of stress resilience, i.e., PLSR resilient functioning scores, as well as versatile opportunities of psychological network analysis (51, 52). Quantifying resilient functioning scores as PLSR residuals is particularly noteworthy as it extends the existing “residual approach” (11). The use of partial least squares regression is still relatively rare in mental health and psychology research (53) and offers considerable opportunities for scientists even beyond resilience research.

### Limitations

Some imitations should be considered when interpreting the present results. First, our results are limited to global connectivity. This means that they refer to self-efficacy as a whole and do not allow for the interpretation of individual self-efficacy facets. Our goal was to embed the concept of self-efficacy in the larger context of resilience. In our opinion, a single questionnaire item should therefore be given rather little weight in the broader context of resilience and resilience factors. Future studies might consider extending our approach to node-wise connectivity, other types of adversity, or other resilience factors.

Quantifying resilience as regression residuals comes with several mathematical challenges. Most important, resilience as regression residuals also include the error of the regression model. This could interfere with a clear interpretation of these scores as stress resilience. Future studies should consider these carefully before applying the “residual approach.”

We applied the Network Comparison Test, the state-of-the-art method to test psychological networks for differences. However, the NCT has not been validated for ordinal data yet. We treated the ordinal self-efficacy items as Gaussian to run the NCT. This might have caused bias during the permutation process. Therefore, the NCT results have to be seen on an exploratory level.

Finally, our study sample does not allow for generalization to the general public. Participants were largely female undergraduate students and represent only a small part of the general population. Future studies therefore have to replicate findings in a more representative and balanced sample with respect to gender distribution, age, and education, in order to allow generalisability of results.

### Outlook

Forthcoming, studies could elaborate resilience factor networks and the role of their connectivity for stress resilience in greater depth. Since stress resilience changes over time (1, 54), it would be particularly interesting to examine the prognostic value of network connectivity for the long-term course of resilience. Such a value as a prognostic indicator has already been attributed to connectivity in the area of depression (30, 31). The corresponding question remains open whether connectivity of resilience factor networks can predict future stress resilience.

It has been shown that self-efficacy and other resilience factors can be increased in cognitive-behavioral interventions (55, 56). Whether connectivity patterns of resilience factors might be useful to inform such interventions requires thorough investigation through longitudinal and causal studies.

In addition, future studies might examine the extent to which our results transfer to other types of adversity and to other resilience factors such as emotion regulation skills or self-esteem. Such studies could include multiple resilience factors into one network, similar to Fritz et al. (15). For research on hybrid networks (2), PLSR residual scores could be used as a node indicative of resilient functioning within the hybrid network.

From a theoretical perspective, the question arises as to why stronger self-efficacy connectivity was associated with higher resilience. We know from psychopathology research that stronger connectivity makes the pathology network more stable overall and less likely to fall back into a healthy state (8, 30). Resilience factors facilitate resilient responses to adversity (54) and our results might indicate that strong connectivity strengthens their effectiveness like thicker strings of a safety net. So, the effectiveness of self-efficacy might once again be enhanced by strong connectivity—with the effect that resilient functioning against stress would as well be promoted by it. Our study provides initial, correlational indications of this in a cross-sectional sample; The theoretical framework would need to be investigated further in the future, e.g., by using longitudinal study designs.

## Conclusion

In this study, we were able to show that in healthy young adults, higher stress resilience is associated with stronger network connectivity of self-efficacy and higher self-efficacy ratings. Our findings transfer results from network research on psychopathology into the field of resilience research–although a deeper understanding of our results has to be gained by future studies. The present study additionally offers an extension of existing methods to calculate a residual-based resilience score by means of Partial Least Squares Regression. Overall, our findings provide evidence for incorporating network approaches, and connectivity patterns in particular, into the study of resilience to stress and resilience factors.

## Data Availability Statement

The raw data supporting the conclusions of this article will be made available by the authors, without undue reservation.

## Ethics Statement

The studies involving human participants were reviewed and approved by Ethics Committee of the Institute of Psychology, Johannes Gutenberg-University Mainz. The patients/participants provided their written informed consent to participate in this study.

## Author Contributions

MW, ES, and KS: study conceptualization. KS, JF, LD, and A-LH: methodology. KS and LD: software and formal analysis. ES: investigation. ES and KS: data curation. KS and JF: writing—original draft preparation. MW, ES, KS, JF, LD, and A-LH: writing—review and editing. KS: visualization. MW: supervision, project administration, and funding acquisition. All authors approved the submitted version. All authors contributed to the article and approved the submitted version.

## Funding

This research was funded by the German Research Foundation (DFG): CRC 1193 Neurobiology of Resilience to Stress-Related Mental Dysfunction (Project C07).

## Conflict of Interest

The authors declare that the research was conducted in the absence of any commercial or financial relationships that could be construed as a potential conflict of interest.

## Publisher's Note

All claims expressed in this article are solely those of the authors and do not necessarily represent those of their affiliated organizations, or those of the publisher, the editors and the reviewers. Any product that may be evaluated in this article, or claim that may be made by its manufacturer, is not guaranteed or endorsed by the publisher.
