# Supplementary material for: Psychological Network Analysis of General Self-Efficacy in High vs. Low Resilient Functioning Healthy Adults
Source: Front Psychiatry. 2021 Nov 17;12:736147. doi: 10.3389/fpsyt.2021.736147 (PMC8635703; doi:10.3389/fpsyt.2021.736147)
Supplement: Supplementary file 1 [file Data_Sheet_1.pdf]

Supplemental Material

List of minor stressors

Here is a complete list of minor stressors that were included in the partial least squares model.

1. Loss or misplacement of items
2. Negative event in the media
3. Negative political event
4. Social obligation
5. Interruption in an activity
6. Waiting time or delay
7. Careless mistake or error due to lack of attention
8. Talk or blasphemy from other people
9. Discrimination or bullying by another person
10. Nightmares
11. Travel/ commuting to work/training place/ school/university
12. Minor offence (e.g. fine for misdemeanour)
13. Inconvenience with authority, office or other institution
14. Conflict or disagreement in the workplace
15. Conflict or disagreement with related parties
16. Conflict or disagreement between related parties
17. Conflict or disagreement with other unrelated persons
18. Conflict or disagreement with your child/children
19. Problem with childcare
20. Procurement or transport service for others
21. Problem or inconvenience caused by your friends or relatives living too far away
22. Problem due to lack of support or help from others

23. Problem with your pet
24. Interference from unsafe environment
25. Impairment by dirt, contamination or stench
26. Problem due to not enough money available
27. Others owe you money
28. You owe money to others
29. High unexpected financial burden
30. Financial affairs
31. Unexpected or unwanted visit
32. Side effect of medication
33. Own physical complaint
34. Physical complaint
35. Lack of sleep or sleep problems
36. Doctor’s visit
37. Office or clerical work at home
38. Housekeeping
39. Performing a minor repair
40. Problem with a technical device
41. Maintenance or servicing of an item
42. Unpleasant or bad weather
43. Interfering behaviour or misconduct of others
44. Bad food
45. Noise
46. Traffic jam
47. Search for a parking space
48. Problem with a communication medium

49. Performance situation at work/school/study
50. High performance requirements or high workload in work/school/study
51. Boring activity
52. Meeting
53. Unregulated or excessive working hours
54. Problem with the planning or arrangement of appointments
55. Time pressure
56. Bad news
57. Problem or inconvenience caused by searching for a training/study/ or job
58. Problem or inconvenience due to house hunting or moving

List of majors stressors

The following shows a full list of major stressors, i.e. significant life events that we used for the PLS model.

1. Job loss
2. Traumatic event at work
3. Marriage plans
4. House purchase, sale or removal
5. Major house renovation
6. Committing a theft or vandalism
7. Violations of the law
8. Serious financial problems
9. Serious illness, accident or diagnosis of illness by you or a close family member
10. Serious illness, accident or diagnosis of a close friend
11. Death of a family member
12. Death of a friend
13. Death of a beloved pet
14. Parental separation
15. Outstanding disputes between family members
16. Separation from boyfriend/girlfriend/partner/ spouse
17. Serious disputes with boyfriend/ girlfriend/ partner/ spouse
18. Serious problems in friendships
19. Start of school of a child
20. Intensive care of an old or sick person
21. Abortion at your or your partner’s home
22. Serious physical illness - unable to work or perform daily activities
23. Injury - unable to work or engage in everyday activities

24. Pregnancy complications or miscarriage of you or your partner
25. Survival of physical abuse or robbery
26. Survival of sexual abuse
27. Any other adverse events (tests, car accident, house fire, earthquake, military action)

Supplementary Figures

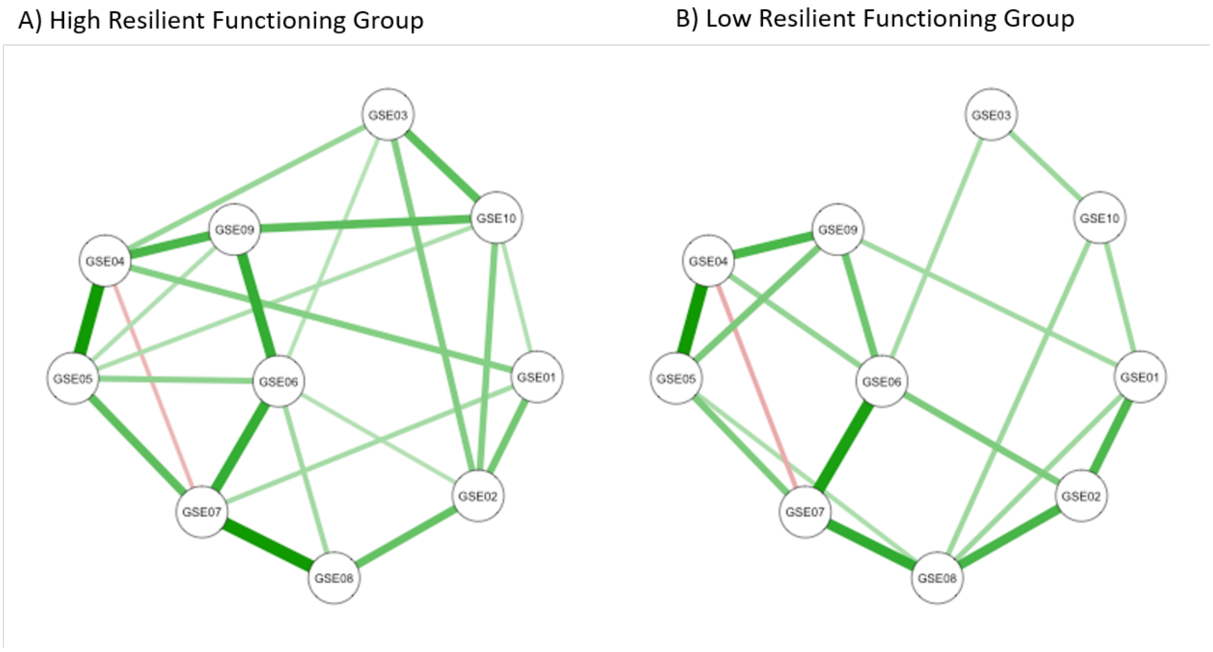

Figure S1: Results of the Network Comparison Test: These self-efficacy networks result from the network comparison test performed on bootnet partial correlation matrices (R package). Data were ordinally scaled. To the best of our knowledge, the NCT has not yet been formally evaluated on ordinal data. **(A)** Left network: high resilient functioning group, **(B)** Right network: low resilient functioning group.

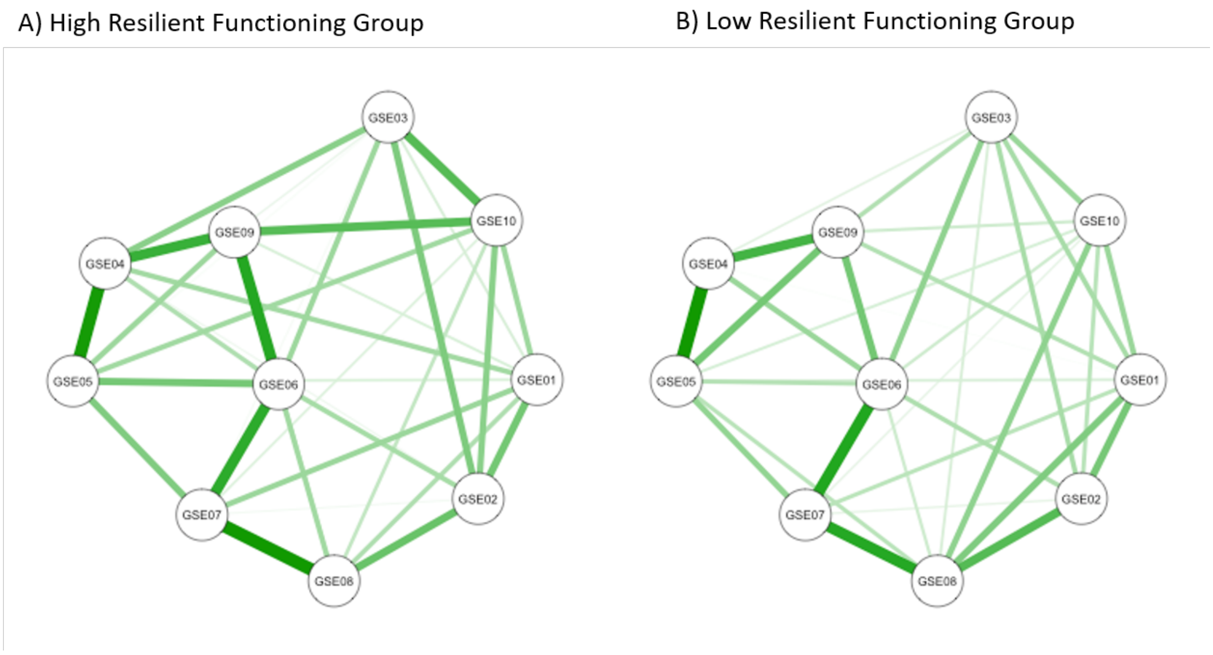

Figure S2: Results of the Network Comparison Test: These self-efficacy networks result from the network comparison test performed on raw data. Data were ordinally scaled. To the best of our knowledge, the NCT has not yet been formally evaluated on ordinal data. **(A)** Left network: high resilient functioning group, **(B)** Right network: low resilient functioning group.
